# Association of Fish Consumption and Mercury Exposure During Pregnancy With Metabolic Health and Inflammatory Biomarkers in Children

**DOI:** 10.1001/jamanetworkopen.2020.1007

**Published:** 2020-03-16

**Authors:** Nikos Stratakis, David V. Conti, Eva Borras, Eduardo Sabido, Theano Roumeliotaki, Eleni Papadopoulou, Lydiane Agier, Xavier Basagana, Mariona Bustamante, Maribel Casas, Shohreh F. Farzan, Serena Fossati, Juan R. Gonzalez, Regina Grazuleviciene, Barbara Heude, Lea Maitre, Rosemary R. C. McEachan, Ioannis Theologidis, Jose Urquiza, Marina Vafeiadi, Jane West, John Wright, Rob McConnell, Anne-Lise Brantsaeter, Helle-Margrete Meltzer, Martine Vrijheid, Leda Chatzi

**Affiliations:** 1 Department of Preventive Medicine, Keck School of Medicine, University of Southern California, Los Angeles; 2 Department of Complex Genetics and Epidemiology, CAPHRI School for Public Health and Primary Care, University of Maastricht, Maastricht, the Netherlands; 3 Universitat Pompeu Fabra, Barcelona, Spain; 4 Proteomics Unit, Centre de Regulacio Genomica, Barcelona Institute of Science and Technology, Barcelona, Spain; 5 Department of Social Medicine, Faculty of Medicine, University of Crete, Heraklion, Crete, Greece; 6 Department of Environmental Health, Norwegian Institute of Public Health, Oslo, Norway; 7 Team of Environmental Epidemiology Applied to Reproduction and Respiratory Health, Inserm, CNRS, University Grenoble Alpes, Institute for Advanced Biosciences, U1209 Joint Research Center, La Tronche, Grenoble, France; 8 Institute for Global Health, Barcelona, Spain; 9 Consorcio de Investigacion Biomedica en Red de Epidemiologia y Salud Publica, Madrid, Spain; 10 Department of Environmental Sciences, Vytautas Magnus University, Kaunas, Lithuania; 11 Centre of Research in Epidemiology and Statistics, Inserm, Institut National de la Recherche Agronomique, Universite de Paris, Paris, France; 12 Bradford Institute for Health Research, Bradford Teaching Hospitals NHS Foundation Trust, Bradford, UK; 13 Foundation for Research and Technology, Institute of Molecular Biology and Biotechnology, Heraklion, Greece

## Abstract

**Importance:**

The balance of mercury risk and nutritional benefit from fish intake during pregnancy for the metabolic health of offspring to date is unknown.

**Objective:**

To assess the associations of fish intake and mercury exposure during pregnancy with metabolic syndrome in children and alterations in biomarkers of inflammation in children.

**Design, Setting, and Participants:**

This population-based prospective birth cohort study used data from studies performed in 5 European countries (France, Greece, Norway, Spain, and the UK) between April 1, 2003, and February 26, 2016, as part of the Human Early Life Exposome (HELIX) project. Mothers and their singleton offspring were followed up until the children were aged 6 to 12 years. Data were analyzed between March 1 and August 2, 2019.

**Exposures:**

Maternal fish intake during pregnancy (measured in times per week) was assessed using validated food frequency questionnaires, and maternal mercury concentration (measured in micrograms per liter) was assessed using maternal whole blood and cord blood samples.

**Main Outcomes and Measures:**

An aggregate metabolic syndrome score for children was calculated using the *z* scores of waist circumference, systolic and diastolic blood pressures, and levels of triglyceride, high-density lipoprotein cholesterol, and insulin. A higher metabolic syndrome score (score range, −4.9 to 7.5) indicated a poorer metabolic profile. Three protein panels were used to measure several cytokines and adipokines in the plasma of children.

**Results:**

The study included 805 mothers and their singleton children. Among mothers, the mean (SD) age at cohort inclusion or delivery of their infant was 31.3 (4.6) years. A total of 400 women (49.7%) had a high educational level, and 432 women (53.7%) were multiparous. Among children, the mean (SD) age was 8.4 (1.5) years (age range, 6-12 years). A total of 453 children (56.3%) were boys, and 734 children (91.2%) were of white race/ethnicity. Fish intake consistent with health recommendations (1 to 3 times per week) during pregnancy was associated with a 1-U decrease in metabolic syndrome score in children (β = −0.96; 95% CI, −1.49 to −0.42) compared with low fish consumption (<1 time per week) after adjusting for maternal mercury levels and other covariates. No further benefit was observed with fish intake of more than 3 times per week. A higher maternal mercury concentration was independently associated with an increase in the metabolic syndrome score of their offspring (β per 2-fold increase in mercury concentration = 0.18; 95% CI, 0.01-0.34). Compared with low fish intake, moderate and high fish intake during pregnancy were associated with reduced levels of proinflammatory cytokines and adipokines in children. An integrated analysis identified a cluster of children with increased susceptibility to metabolic disease, which was characterized by low fish consumption during pregnancy, high maternal mercury levels, decreased levels of adiponectin in children, and increased levels of leptin, tumor necrosis factor α, and the cytokines interleukin 6 and interleukin 1β in children.

**Conclusions and Relevance:**

Results of this study suggest that moderate fish intake consistent with current health recommendations during pregnancy was associated with improvements in the metabolic health of children, while high maternal mercury exposure was associated with an unfavorable metabolic profile in children.

## Key Points

**Question:**

Is fish consumption during pregnancy associated with benefits for the metabolic health of children?

**Findings:**

In this cohort study of 805 mothers and their singleton offspring, moderate fish consumption during pregnancy was associated with the downregulation of inflammation and improvements in the metabolic profile of children; high mercury exposure during pregnancy had the opposite associations.

**Meaning:**

The results of this study suggest that fish consumption consistent with current recommendations during pregnancy was associated with improvements in the metabolic health of children.

## Teaser

This cohort study used data from the Human Early Life Exposome (HELIX) project, a collaboration of 5 European birth cohort studies, to examine the associations of maternal fish consumption and mercury exposure during pregnancy with metabolic health and inflammatory biomarkers in children.

## Introduction

Cardiovascular disease, a common cause of mortality worldwide,^1^ has its origins in early life.^2^ Traditional cardiometabolic risk markers, including central obesity, high blood pressure (BP), dyslipidemia, and hyperinsulinemia, share common pathophysiological mechanisms, including inflammation, and are likely to not only coexist in childhood but to continue into adulthood.^3,4^ Therefore, it is important to identify early determinants of risk that can be targeted for preventive interventions.

Fish is the major dietary source of ω-3 long-chain polyunsaturated fatty acids, which may have anti-inflammatory properties and may be associated with cardiometabolic benefits.^5,6^ However, fish is also a common source of exposure to mercury, which may be associated with opposing consequences.^7,8,9,10^ Adult studies suggest that moderate fish consumption is associated with a lower risk of cardiovascular disease, with little or no further benefit observed with fish intake of more than 3 times per week.^7,11^ Nevertheless, to date, the extent to which prenatal fish intake is associated with the metabolic health of children remains uncertain. Prenatal biomarker levels or the intake of ω-3 long-chain polyunsaturated fatty acids were associated with a better cardiometabolic profile among children in some, but not all, studies.^12,13^ One birth cohort study examined fish intake during pregnancy and reported an association only among women with gestational diabetes.^14^

Most previous studies did not consider concomitant mercury exposure, which may at least partially attenuate the benefits of fish consumption and account for the inconsistent findings. Cross-sectional data in studies of children have associated mercury exposure with greater adiposity and increased cholesterol levels.^15,16^ The limited investigations of maternal mercury exposure and the cardiometabolic health of their offspring have focused primarily on blood pressure (BP), with reports of positive (ie, increased BP)^17,18^ or null^19,20^ associations.

In this study, we aimed to (1) assess the associations between maternal fish intake, mercury exposure during pregnancy, and the metabolic profile of offspring; (2) examine whether maternal fish intake and mercury exposure are associated with concentrations of inflammatory cytokines and adipokines in the plasma of children; and (3) identify clusters of children with increased susceptibility to metabolic disease by integrating fish intake and mercury exposure during pregnancy and markers of inflammation in children using an innovative latent variable analysis.

## Methods

Approval for the Human Early Life Exposome (HELIX) project was obtained from the local ethics committees at each site. In addition, our study was approved by the institutional review board of the University of Southern California, Los Angeles. All participating families provided written informed consent. Our study followed the Strengthening the Reporting of Observational Studies in Epidemiology (STROBE) reporting guideline for cohort studies.^21^

### Study Population

We used data from the HELIX project,^22^ which was a collaboration of the following European population-based birth cohort studies: (1) the Born in Bradford (BiB) study (Bradford, UK); (2) the Etude des Determinants Pre et Postnatals du Developpement et de la Sante de l’Enfant (EDEN) study (Poitiers, France); (3) the Kaunas Newborn Cohort (KANC) study (Kaunas City, Lithuania); (4) the Infancia y Medio Ambiente (INMA) study (Sabadell, Spain); (5) the Norwegian Mother, Father and Child (MoBa) study (Norway)^23^; and (6) the Rhea Mother-Child (RHEA) study (Herakion, Greece). The studies were conducted between April 1, 2003, and February 26, 2016, and the total recruitment period across cohorts was from April 1, 2003, to January 30, 2009. Our study population consisted of 805 mothers and their singleton children, with information on fish intake and mercury exposure obtained during pregnancy, and complete data on the cardiometabolic factors and protein concentrations of children obtained during follow-up between December 1, 2013, and February 26, 2016. Mothers and their singleton offspring were followed up until the children were aged 6 to 12 years.

### Fish Intake and Mercury Concentration

Information on maternal fish intake was derived from cohort-specific food frequency questionnaires (eTable 1 in the Supplement).^24^ The KANC cohort had no information on maternal diet and was therefore not included in our analysis. As in previous work,^25^ we categorized fish intake into low (<1 time per week), moderate (1-3 times per week, consistent with recommendations from the US Food and Drug Administration and the Environmental Protection Agency),^26^ and high (>3 times per week).

Mercury levels were assessed in maternal whole blood samples using inductively coupled plasma mass spectrometry or in cord blood samples using thermal decomposition, amalgamation, and atomic absorption spectrometry (eMethods 1 in the Supplement). Cord blood concentrations were divided by 1.7 to be comparable with whole blood concentrations.^27^

### Adiposity, Lipid Levels, and Blood Pressure

Waist circumference and BP were measured in children using a common protocol.^22^ High-density lipoprotein (HDL) cholesterol and triglyceride levels were assessed in serum using homogeneous enzymatic colorimetric methods in the MODULAR *ANALYTICS* system (Roche Diagnostics) according to the manufacturer’s instructions. Insulin levels were assessed using the human adipokine 15-plex magnetic panel (Life Technologies) (eMethods 2 in the Supplement).

We constructed *z* scores for the waist circumference, systolic and diastolic BPs, triglyceride level, HDL cholesterol level, and insulin level of children using sex and age standardization.^28^ For BP, we also standardized for height.^28^ Triglyceride levels had a skewed distribution and were log-transformed before analysis. Our primary outcome of interest was a continuous metabolic syndrome score built according to the following formula: metabolic syndrome = z waist circumference + (–z HDL cholesterol level + z triglyceride level)/2 + z insulin + (z systolic BP + z diastolic BP)/2.

This score was used to reflect a metabolic health profile, with a higher score indicating a poorer profile. This scoring system has been previously validated in the European multicenter Identification and Prevention of Dietary and Lifestyle-Induced Health Effects in Children and Infants (IDEFICS) study.^28^

### Inflammatory Cytokines and Adipokines

Three protein panels, the human cytokine 30-plex magnetic panel, the human apolipoprotein 5-plex magnetic panel, and the human adipokine 15-plex magnetic panel (Life Technologies), were used to measure the plasma of children at the University Pompeu Fabra Centre for Genomic Regulation Proteomics Unit (Barcelona, Spain) using the Luminex xMAP multiplex platform (Luminex Corp) (eMethods 2 in the Supplement).

Using previous knowledge from the Kyoto Encyclopedia of Genes and Genomes database, we grouped proteins into 5 pathways that are considered to play key roles in inflammation and cardiometabolic health: the Janus kinase signal transducer and activator of transcription proteins signaling pathway,^29^ the adipocytokine signaling pathway,^30^ the cholesterol metabolism pathway,^31^ the nuclear factor-kappa B pathway,^32^ and the chemokine signaling pathway.^33^

### Covariates

The selection of covariates for adjustment (eMethods 3 in the Supplement) was based on previous studies and a directed acyclic graph approach (eFigure 1 in the Supplement).

We included the following covariates in the models: maternal age (in years), maternal and paternal educational level (low, middle, and high), maternal prepregnancy body mass index (calculated as weight in kilograms divided by height in meters squared), maternal parity (primiparous, defined as having 1 pregnancy only, and multiparous, defined as having ≥2 pregnancies), and the children’s race/ethnicity (white, Asian, and other).

### Statistical Analysis

#### Health Outcomes, Proteins, and Pathways

We assessed the associations of fish intake and mercury levels during pregnancy with the metabolic syndrome score of children as well as with the individual components of the metabolic syndrome score as secondary outcomes. Mercury concentration (measured in micrograms per liter) was log_2_-transformed for normality and then treated as a continuous variable because no departures from linearity in the associations between mercury levels and health outcomes were observed, either visually or statistically (*P* for linearity >.28), using generalized additive models. We performed regression models that included separate maternal fish intake and mercury levels. We then included both fish intake and mercury levels in the models to assess their independent associations with the health outcomes of children. We also included a product term between fish intake and mercury levels in the regression analysis to assess interaction. To simplify interpretation of this model, we categorized maternal mercury levels as high and low based on the cutoff of 3.5 μg/L, which is considered the level of concern for a developing fetus.^34^ In all models, we included a cohort indicator as a fixed effect because doing so in the context of an observational study can provide unbiased control of the cohort effects.^35^ We imputed missing values for covariates using the multivariate imputation by chained equations (MICE) package in R software (R Foundation).^36^ We performed analyses with both original (total missingness, 8.8% in covariates) and imputed data. The results were similar; hence, we presented those findings using the imputed covariate data.

We performed several sensitivity analyses to assess the robustness of our results. First, we assessed between-cohort heterogeneity by computing the *I*^2^ statistic.^37^ Second, we repeated the analysis while excluding 1 cohort at a time. Third, we assessed the effect modification by maternal educational level (low/middle or high), gestational diabetes status (yes or no), and sex of the children by testing the interactions between the potential effect modifiers and the maternal fish intake or mercury levels. Based on previous studies, we hypothesized that the exposure-outcome associations would be stronger among girls^25^ and children whose mothers had a lower educational level^38^ or gestational diabetes.^14^ Fourth, we further adjusted for gestational weight gain, available food indicators of maternal diet quality (consumption of fruits, vegetables, cereals, and fast food), breastfeeding, and the sedentary behavior, mercury concentration, and diet (consumption of fast food, sugar-sweetened beverages, sweets, and fish intake) of the children. Fifth, we also adjusted for maternal blood levels of environmental pollutants (polychlorinated biphenyls and dichlorodiphenyldichloroethylene) and arsenic.^7,39^

We then examined the associations of maternal fish intake and mercury concentration with the protein levels of the children using linear regression models adjusted for the same covariates plus the age and sex of the children. We subsequently employed a second-stage hierarchical model to calculate pathway-level effect estimates by modeling each individual protein as a linear function of the Kyoto Encyclopedia of Genes and Genomes pathway indicator variables (distinguishing whether a protein does or does not belong to a prespecified pathway) using an inverse-variance weight.^40^ This approach allowed us to estimate an overall effect for each pathway (ie, the effect estimate of exposure on a protein within a specific pathway).

#### Integrated Analysis

We performed an integrated latent variable analysis to identify clusters of children associated with increased susceptibility to metabolic disease based on variables including maternal fish intake, mercury exposure during pregnancy, and the children’s protein profile by using the latent unknown clustering with integrated data (LUCIDus) package in R software (R Foundation).^41^ We obtained effect estimates for the association of estimated latent clusters with the metabolic syndrome score (eMethods 4 in the Supplement). Proteins were selected if they were significantly associated with the exposures of interest at a significance level of *P* < .05.

All data analyses were conducted using Stata software, version 14.2 (StataCorp), and R software, version 3.5.3 (R Foundation). Data were analyzed between March 1 and August 2, 2019.

## Results

### Participant Characteristics

Among 805 mothers in the analysis, the mean (SD) age at cohort inclusion or delivery of an infant was 31.3 (4.6) years (Table 1). A total of 400 women (49.7%) had a high educational level, and 432 women (53.7%) were multiparous. The mean (SD) fish intake during pregnancy was 3.7 (3.3) times per week. Compared with women with low fish intake (n = 117), women with moderate and high fish intake (n = 317 and n = 371, respectively) were more likely to be older (mean [SD] age, 30.6 [4.8] years among women with low intake vs 30.9 [5.0] years among women with moderate intake and 31.9 [4.2] years among women with high intake) and to have a high educational level (51 women [43.6%] with low intake vs 155 women [48.9%] with moderate intake and 194 women [52.3%] with high intake) (eTable 2 in the Supplement). The median (interquartile range) concentration of maternal blood mercury was 2.5 (1.5-4.2) μg/L. Mercury concentrations were modestly associated with fish consumption during pregnancy (Spearman *r* = 0.2). Women with high fish consumption were more likely to have mercury concentrations equal to or greater than the level of concern given as 3.5 μg/L (155 women [41.8%] with high intake vs 36 women [30.8%] with low intake and 79 women [24.9%] with moderate intake) (eTable 2 in the Supplement).

**Table 1.** Characteristics of Study Population^a^

Characteristic	No. (%) (N = 805)
Cohort	
BiB, UK	104 (12.9)
EDEN, France	143 (17.8)
INMA, Spain	204 (25.3)
MoBa, Norway	211 (26.2)
RHEA, Greece	143 (17.8)
Parent	
Maternal fish intake during pregnancy, mean (SD), times/wk	3.7 (3.3)
Frequency of fish intake during pregnancy	
Low (<1 time/wk)	117 (14.5)
Moderate (≥1 to ≤3 times/wk)	317 (39.4)
High (>3 times/wk)	371 (46.1)
Maternal age, mean (SD), y	31.3 (4.6)
Missing, No. (%)	3 (0.4)
Maternal prepregnancy BMI, mean (SD)	24.0 (4.4)
Maternal prepregnancy weight	
Normal (BMI<25)	552 (68.6)
Overweight (BMI≥25)	243 (30.2)
Missing	10 (1.2)
Maternal smoking during pregnancy	
No	662 (82.2)
Yes	131 (16.3)
Missing	12 (1.5)
Gestational diabetes status	
No	384 (47.7)
Yes	41 (5.1)
Missing	380 (47.2)
Maternal parity	
Primiparous	368 (45.7)
Multiparous	432 (53.7)
Missing	5 (0.6)
Maternal educational level	
Low	106 (13.2)
Medium	283 (35.2)
High	400 (49.7)
Missing	16 (2.0)
Paternal educational level	
Low	130 (16.1)
Medium	294 (36.5)
High	331 (41.1)
Missing	50 (6.2)
Maternal mercury concentration, median (interquartile range), μg/L	2.5 (1.5-4.2)
Child	
Age at assessment, mean (SD), y	8.4 (1.5)
Sex	
Male	453 (56.3)
Female	352 (43.7)
Birth weight, mean (SD), g	3347 (488)
Gestational age, mean (SD), wk	39.7 (1.7)
Race/ethnicity	
White	734 (91.2)
Asian	55 (6.8)
Other	16 (2.0)
Waist circumference, mean (SD), cm	59.3 (7.7)
HDL cholesterol level, mean (SD), mg/dL	60.1 (12.5)
Triglyceride level, median (interquartile range), mg/dL	75.3 (58.5-99.2)
Insulin level, median (interquartile range), μg/mL	5.5 (4.2-8.5)
Blood pressure, mean (SD), mm Hg	
Systolic	100.5 (10.6)
Diastolic	58.2 (9.4)
Metabolic syndrome score, mean (SD)^b^	−0.1 (2.3)

Abbreviations: BiB, Born in Bradford study; BMI, body mass index (calculated as weight in kilograms divided by height in meters squared); EDEN, Etude des Determinants Pre et Postnatals du Developpement et de la Sante de l’Enfant study; HDL, high-density lipoprotein; INMA, Infancia y Medio Ambiente study; MoBa, Norwegian Mother, Father and Child study; RHEA, Rhea Mother-Child study.

SI conversion factors: To convert HDL cholesterol from mg/dL to mmol/L, multiply by 0.0259; triglycerides from mg/dL to mmol/L, multiply by 0.0113; and insulin from micromoles per milliliter to picomoles per liter, multiply by 6.945.

^a^ Continuous data are presented as means (SDs) if normally distributed or as medians (interquartile range) if not normally distributed.

^b^ The metabolic syndrome score (expressed in mean [SD]) was derived using *z* scores for waist circumference, HDL cholesterol level, triglyceride level, insulin level, and systolic and diastolic blood pressure.

Among 805 children in the study, the mean (SD) age was 8.4 (1.5) years (age range, 6-12 years). A total of 453 children (56.3%) were boys, and 734 children (91.2%) were of white race/ethnicity. Additional data regarding mercury exposure and other characteristics of the study population are shown in Table 1.

### Fish Intake, Mercury Exposure, and Metabolic Syndrome

Compared with low fish intake during pregnancy, moderate fish intake was associated with a 1-U decrease in the metabolic syndrome score of children (β = −0.96; 95% CI, −1.49 to −0.42) (*P* < .001), while high fish intake was associated with a smaller 0.7-U decrease in the metabolic syndrome score (β, −0.71; 95% CI, −1.33 to −0.10; *P* = .02; Table 2). A doubling in maternal blood mercury concentration was associated with a 0.18 higher metabolic syndrome score (β = 0.18; 95% CI, 0.01-0.34; *P* = .03). Inclusion of maternal fish intake and mercury exposure in the same model slightly strengthened the effect estimates, especially for high fish intake. Compared with low fish intake and low mercury exposure, the combination of moderate fish consumption and low mercury exposure was associated with the greatest decrease in metabolic syndrome score (β = −0.70; 95% CI, −1.33 to −0.07; *P* = .03), while the combination of low fish consumption and high mercury exposure was associated with the greatest increase in metabolic syndrome score (β = 0.93; 95% CI, 0.06-1.79; *P* = .04). However, the interaction between fish intake and mercury exposure was not significant (*P* = .32).

**Table 2.** Association of Fish Intake During Pregnancy and Maternal Mercury Levels With Metabolic Syndrome Score Among Children Aged 8 Years

Model	Metabolic Syndrome Score (N = 805)^a^	
	β Estimate (95% CI)	*P* Value
Separate models		
Fish intake, times/wk		
<1 [Reference]	NA	NA
≥1 to ≤3	−0.92 (−1.45 to −0.38)	.001
>3	−0.59 (−1.20 to 0.02)	.06
Mercury exposure, log_2_ μg/L	0.17 (0.01 to 0.33)	.04
Mutually adjusted model		
Fish intake, times/wk		
<1 [Reference]	NA	NA
≥1 to ≤3	−0.96 (−1.49 to −0.42)	<.001
>3	−0.71 (−1.33 to −0.10)	.02
Mercury exposure, log_2_ μg/L	0.18 (0.01 to 0.34)	.03
Effect heterogeneity^b^		
Fish intake, times/wk (mercury exposure, μg/L)		.32^c^
<1 (<3.5) [Reference]	NA	NA
<1 (≥3.5)	0.93 (0.06 to 1.79)	.04
1-3 (<3.5)	−0.70 (−1.33 to −0.07)	.03
1-3 (≥3.5)	−0.56 (−1.27 to 0.16)	.13
>3 (<3.5)	−0.51 (−1.20 to 0.18)	.15
>3 (≥3.5)	−0.06 (−0.79 to 0.67)	.87

Abbreviation: NA, not applicable.

^a^ The metabolic syndrome score is expressed in mean (SD) and was derived using *z* scores for waist circumference, high-density lipoprotein cholesterol level, triglyceride level, insulin level, and systolic and diastolic blood pressure. Estimates are β coefficients (95% CIs) calculated by linear regression models that were adjusted for maternal age, maternal prepregnancy body mass index (calculated as weight in kilograms divided by height in meters squared), parental education, maternal parity, children’s race/ethnicity, and cohort.

^b^ The following numbers of participants were included for each combination of fish intake and mercury exposure: 80 participants for low fish intake (<1 time/week) and low mercury exposure (<3.5 μg/L); 37 participants for low fish intake and high mercury exposure (≥3.5 μg/L); 238 participants for moderate fish intake (1-3 times per week) and low mercury exposure; 79 participants for moderate fish intake and high mercury exposure; 216 participants for high fish intake (>3 times per week) and low mercury exposure; and 155 participants for high fish intake and high mercury exposure.

^c^ *P* value for interaction between fish intake and mercury exposure.

**Figure 1.**
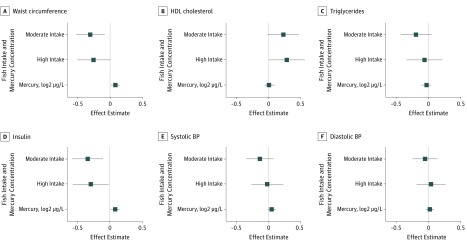
Association of Fish Intake During Pregnancy and Maternal Mercury Levels With Metabolic Risk Factors of Children Aged 8 Years The figure represents data from 805 mothers and their singleton offspring who participated in the Human Early Life Exposome study. Metabolic risk factors are expressed as *z* scores. Effect estimates represent β coefficients (squares) and 95% CIs (error bars) calculated by linear regression models that included maternal fish intake and mercury concentrations and were further adjusted for maternal age, maternal prepregnancy body mass index (calculated as weight in kilograms divided by height in meters squared), parental education, maternal parity, children’s race/ethnicity, and cohort. For fish intake, the reference category was low intake (<1 time per week). Moderate fish intake was defined as 1 to 3 times per week. High fish intake was defined as more than 3 times per week. BP indicates blood pressure; HDL, high-density lipoprotein.

In analyses examining the individual components of the metabolic syndrome score, we found that maternal fish intake was most strongly inversely associated with the waist circumference and insulin level of children and was positively associated with the HDL cholesterol level of children (Figure 1). Maternal mercury exposure was positively associated with the waist circumference and insulin level of children.

In sensitivity analyses, we did not find evidence of between-cohort heterogeneity (*I*^2^ = 0% for all estimates), and the results remained similar after the exclusion of 1 cohort at a time (eFigure 2 in the Supplement). We also did not find indications that the associations differed by the sex of the child, the maternal educational level, or the gestational diabetes status (eTable 3 in the Supplement). The results did not materially change when we also adjusted for dietary and lifestyle factors among mothers and children (eTable 4 in the Supplement) or maternal exposure to environmental pollutants (eTable 5 in the Supplement).

### Fish Intake, Mercury Exposure, and Inflammatory Biomarkers

**Figure 2.**
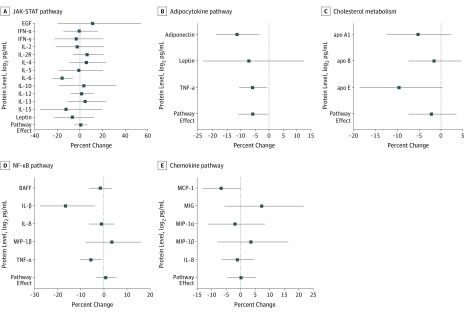
Association of Fish Intake During Pregnancy With Child Protein Levels at Age 8 Years The figure represents data from 805 mothers and their singleton offspring who participated in the Human Early Life Exposome study. Effect estimates represent percentage changes in protein levels expressed as log_2_ picograms per milliliter (squares) and their 95% CIs (error bars) for fish intake of equal to or more than 1 time per week compared with less than 1 time per week. Models were adjusted for maternal mercury concentrations, maternal age, maternal prepregnancy body mass index (calculated as weight in kilograms divided by height in meters squared), parental education, maternal parity, children’s race/ethnicity, and cohort. apo indicates apolipoprotein; BAFF, B-cell activating factor of the tumor necrosis factor (TNF) family; EGF, epidermal growth factor; IFN, interferon; IL, interleukin; JAK-STAT, Janus kinase signal transducer and activator of transcription proteins signaling pathway; MCP, methyl-accepting chemotaxis protein; MIG, CXC chemokine 9; MIP, macrophage inflammatory protein; NF-κB, nuclear factor–kappa B; and TNF-α, tumor necrosis factor α.

Compared with low maternal fish intake, moderate and high fish intake were associated with lower levels of the cytokines interleukin (IL) 1β (IL-1β; percentage change, −16.7%; 95% CI, −27.9% to −3.9%; *P* = .01) and IL-6 (percentage change, −16.2%; 95%, CI, −24.9% to −6.6%; *P* = .001), adiponectin (percentage change, −11.4%; 95% CI, −18.7% to −3.4%; *P* = .01), and tumor necrosis factor α (TNF-α) (percentage change, −5.7%; 95% CI, −10.5% to −0.6%; *P* = .03) (Figure 2; eTable 6 in the Supplement). There was also a decrease in apolipoprotein E levels associated with moderate and high fish intake compared with low fish intake (percentage change, −9.7%; 95% CI, −18.7% to −0.3%; *P* = .06). Maternal mercury blood concentrations were not associated with protein levels in children (eTable 6 in the Supplement). The hierarchical pathway analysis indicated that moderate and high fish intake during pregnancy were associated with an average decrease of 5.7% in protein levels (95% CI, −10.80% to −0.32%; *P* = .04) within the adipocytokine pathway (Figure 2; eTable 6 in the Supplement).

### Integrated Analysis

**Figure 3.**
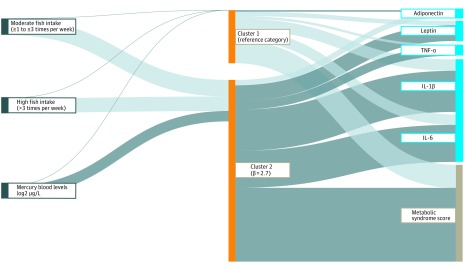
Integrated Analysis of Fish Intake During Pregnancy, Maternal Mercury Levels, and Individual Protein Levels of Children for the Identification of a Subgroup of Children With Poorer Metabolic Health The thick light gray lines connecting fish intake categories to cluster 2 indicate negative associations. The thick dark gray line connecting mercury to cluster 2 indicates a positive association. The dark gray lines connecting the clusters to proteins indicate positive associations, and the light gray lines suggest negative associations. The width of the lines is proportional to the effect size. The thick gray line connecting cluster 2 and metabolic syndrome score indicates that children in the latent cluster 2 had a higher metabolic syndrome score compared with children in cluster 1. The metabolic syndrome score is expressed in SD and was derived using *z* scores for waist circumference, high-density lipoprotein cholesterol level, triglyceride level, insulin level, and systolic and diastolic blood pressure. IL-1β indicates interleukin 1β; IL-6, interleukin 6; and TNF-α, tumor necrosis factor α.

In the integrated latent analysis, we identified 2 clusters of children. Cluster 2 was defined as the high-risk cluster, which was characterized by a 2.7-U higher metabolic syndrome score compared with cluster 1 (the reference cluster; Figure 3). Cluster 2 was negatively associated with maternal fish intake and positively associated with maternal mercury levels. This cluster was also characterized by increased levels of leptin, TNF-α, IL-1β, and IL-6 and lower levels of adiponectin. To understand how fish intake, mercury exposure, and protein profiles were associated with the latent cluster estimation, we assigned each child to 1 of the 2 clusters based on an estimated probability greater than 0.5 for membership within a cluster in a post hoc analysis. Children assigned to the high-risk cluster had associations reflective of the proteins that characterized cluster 2 and a poorer cardiometabolic profile than children assigned to cluster 1 (eTable 7 in the Supplement).

## Discussion

The findings of this study suggest that fish intake during pregnancy, especially moderate fish intake that is consistent with current recommendations, is associated with improvements in the metabolic profile of children, as indicated by a lower metabolic syndrome score. Higher mercury exposure during pregnancy was associated with a poorer metabolic profile in children. We also found that fish intake and mercury levels during pregnancy could be used to characterize subgroups of children with alterations in inflammatory cytokines and adipokines and that these subgroups were associated with metabolic health in children; these results are consistent with a role for inflammation in the metabolic consequences of fish intake and mercury exposure.

We used an aggregate metabolic syndrome score as an indicator of overall metabolic dysfunction. Previous research has indicated that the metabolic syndrome score is a useful tool for detecting subclinical atherosclerotic risk based on carotid intima-media thickness and vascular stiffness parameters^42^ and that this risk is likely to continue into adulthood.^43^ Our study suggests that compared with low fish intake, the intake of more than 1 but less than 3 servings of fish per week was associated with a better metabolic syndrome score in children. This better score was primarily owing to beneficial associations with waist circumference, HDL cholesterol levels, and insulin levels. An attenuation of the metabolic benefit was observed with maternal fish intake that was higher than the recommended amount (>3 times per week). Similar to our findings, adult studies have suggested a nonlinear threshold association between fish consumption and the risk of cardiovascular disease, with the highest benefit observed among those with moderate fish consumption levels.^7,11^ Our study did not confirm the findings of a previous Danish study, which suggested that gestational diabetes status may modify the consequences of prenatal fish intake. The smaller number of participants with gestational diabetes in our study compared with the Danish study (5% vs 44.3%, respectively) or diabetes status misclassification may explain the inconsistency in findings.

Concomitant mercury exposure may mask or counterbalance the benefits of fish consumption, especially at high levels of intake. Mercury exposure has been associated with metabolic syndrome, visceral adiposity, and insulin resistance, especially in adults.^44,45,46,47^ We observed an association of prenatal mercury exposure with a higher metabolic syndrome score in children, which was largely owing to positive associations with waist circumference and insulin levels. The effect estimates of mercury exposure were smaller in magnitude but independent of those of maternal fish consumption. To our knowledge, no previous study has examined the association of prenatal mercury exposure with overall metabolic profile or adiposity and insulin measures in children. The adjustment for maternal mercury exposure in our analysis strengthened the effect estimates for the high intake of fish. However, this adjustment did not fully explain the lower metabolic benefit observed with high intake compared with moderate intake. This attenuation in benefit remained even after further adjustment for organic pollutants (polychlorinated biphenyls and dichlorodiphenyldichloroethylene) and arsenic, which are commonly found in fish and might have adverse metabolic effects.^48^

Using a novel integrated analysis, this is the first human study, to our knowledge, to report that alterations in the cytokines TNF-α, IL-6, and IL-1β and the adipokines leptin and adiponectin in response to maternal fish consumption and mercury exposure during pregnancy might be associated with metabolic consequences in children. Experimental evidence suggests that n-3 fatty acids found in fish may be associated with reductions in TNF-α, IL-6, and *IL-1β* gene expression via activation of the G protein–coupled receptor 120.^49^ In contrast, mercury exposure has been reported to activate p38 mitogen-activated protein kinase and alter secretion of these cytokines.^50^ Moreover, ω-3 fatty acids and mercury can alter adipokine secretion and modulate inflammatory response through changes in endoplasmic reticulum stress and the peroxisome proliferator-activated receptor signaling pathway.^6,51^ Further studies are needed to replicate these results and identify the underlying biological mechanisms that may explain the association of prenatal fish intake and mercury exposure with metabolic health in children.

### Strengths and Limitations

The main strengths of the study are the multicentric design, which included mother-child pairs from 5 countries spanning northern to southern Europe, the use of standardized protocols for outcome assessment, the detailed characterization of inflammatory biomarkers in children to gain insight into potential underlying pathways, and the application of a novel integrated analysis for identifying subgroups of children with a poor metabolic profile.

Our study had several limitations. We used self-reported dietary information; hence, maternal fish intake may have been misclassified. Nevertheless, the use of validated food frequency questionnaires and the consistency of the associations across cohorts suggest that misclassification of fish intake is an unlikely explanation for the observed results. Among fish species, there is considerable variation in the content of both ω-3 long-chain polyunsaturated fatty acids, which are considered the most likely active beneficial nutrients, and mercury. However, we did not have information regarding the types of fish consumed or the ω-3 long-chain polyunsaturated fatty acid intake; therefore, it was not possible to examine the associations of fish intake with different nutrient and mercury content. As in any observational study, the possibility of unmeasured residual confounding existed. However, the results did not change when we adjusted our models for a number of dietary and lifestyle factors among mothers and children. In addition, our study population consisted of primarily healthy children, which did not allow us to examine clinical metabolic end points owing to small numbers.

## Conclusions

This study’s findings appear to indicate that fish intake during pregnancy, especially moderate fish intake of 1 or more to 3 or less servings per week, was associated with an improved metabolic profile in offspring. Higher mercury exposure during pregnancy was associated with a poorer metabolic profile. The novel integrated latent variable approach suggested that changes in key inflammatory cytokines and adipokines characterize these associations. Our results suggest that, for pregnant women, the benefit of fish intake that is consistent with recommendations from the US Food and Drug Administration and the Environmental Protection Agency exceeds the risk in terms of the metabolic health of children. We believe the potential metabolic harm of mercury exposure is of concern and that efforts to limit mercury contamination are needed.

## Notes

Supplement. **eMethods 1.** Measurement of Mercury Levels During Pregnancy **eMethods 2.** Measurement of Inflammatory Biomarkers and Adipokines in Children **eMethods 3.** Covariates **eMethods 4.** Structural Integrated Latent Variable Analysis **eTable 1.** Description of Cohort-Specific Food Questionnaires Used to Assess Maternal Diet During Pregnancy **eTable 2.** Comparison of Characteristics of the Study Population (N = 805) by Categories of Maternal Fish Consumption During Pregnancy **eTable 3.** Associations of Fish Intake During Pregnancy and Maternal Mercury Levels With Metabolic Syndrome Score Among Children Aged 8 Years in HELIX After Stratifying for Child Sex, Maternal Education Level, and Gestational Diabetes Status **eTable 4.** Associations of Fish Intake During Pregnancy and Maternal Mercury Levels With Metabolic Syndrome Score Among Children Aged 8 Years in HELIX (N = 805) After Further Adjusting for Gestational Weight Gain, Breastfeeding, Child Sedentary Behavior, Child Mercury Concentration, and a Number of Maternal and Child Food Indicators of Diet Quality **eTable 5.** Associations of Fish Intake During Pregnancy and Maternal Mercury Levels With Metabolic Syndrome Score Among Children Aged 8 Years in HELIX (N = 805) After Further Adjusting for Maternal Plasma Levels of Polychlorinated Biphenyls, Dichlorodiphenyldichloroethylene, and Arsenic **eTable 6.** Associations of Fish Intake During Pregnancy and Maternal Mercury Concentrations With Protein Levels Among Children Aged 8 Years in HELIX (N = 805) **eTable 7.** Distribution of Exposure and Proteins in Subgroups From the Latent Integrated Analysis Defined With Individuals Assigned to the Most Likely Cluster **eFigure 1.** Directed Acyclic Graph on the Association of Maternal Fish Intake During Pregnancy and Metabolic Health in Children **eFigure 2.** Associations of Fish Intake During Pregnancy and Maternal Mercury Levels With Metabolic Syndrome Score Among Children Aged 8 Years in HELIX After Omitting 1 Cohort at a Time **eReferences.**
