# Supplementary material for: Association of Fish Consumption and Mercury Exposure During Pregnancy With Metabolic Health and Inflammatory Biomarkers in Children
Source: JAMA Netw Open. 2020 Mar 16;3(3):e201007. doi: 10.1001/jamanetworkopen.2020.1007 (PMC7076335; doi:10.1001/jamanetworkopen.2020.1007)
Supplement: Supplement. — eMethods 1. Measurement of Mercury Levels During Pregnancy eMethods 2. Measurement of Inflammatory Biomarkers and Adipokines in Children eMethods 3. Covariates eMethods 4. Structural Integrated Latent Variable Analysis eTable 1. Description of Cohort-Specific Food Questionnaires Used to Assess Maternal Diet During Pregnancy eTable 2. Comparison of Characteristics of the Study Population (N = 805) by Categories of Maternal Fish Consumption During Pregnancy eTable 3. Associations of Fish Intake During Pregnancy and Maternal Mercury Levels With Metabolic Syndrome Score Among Children Aged 8 Years in HELIX After Stratifying for Child Sex, Maternal Education Level, and Gestational Diabetes Status eTable 4. Associations of Fish Intake During Pregnancy and Maternal Mercury Levels With Metabolic Syndrome Score Among Children Aged 8 Years in HELIX (N = 805) After Further Adjusting for Gestational Weight Gain, Breastfeeding, Child Sedentary Behavior, Child Mercury Concentration, and a Number of Maternal and Child Food Indicators of Diet Quality eTable 5. Associations of Fish Intake During Pregnancy and Maternal Mercury Levels With Metabolic Syndrome Score Among Children Aged 8 Years in HELIX (N = 805) After Further Adjusting for Maternal Plasma Levels of Polychlorinated Biphenyls, Dichlorodiphenyldichloroethylene, and Arsenic eTable 6. Associations of Fish Intake During Pregnancy and Maternal Mercury Concentrations With Protein Levels Among Children Aged 8 Years in HELIX (N = 805) eTable 7. Distribution of Exposure and Proteins in Subgroups From the Latent Integrated Analysis Defined With Individuals Assigned to the Most Likely Cluster eFigure 1. Directed Acyclic Graph on the Association of Maternal Fish Intake During Pregnancy and Metabolic Health in Children eFigure 2. Associations of Fish Intake During Pregnancy and Maternal Mercury Levels With Metabolic Syndrome Score Among Children Aged 8 Years in HELIX After Omitting 1 Cohort at a Time eReferences. [file jamanetwopen-3-e201007-s001.pdf]

Supplementary Online Content

Stratakis N, Conti DV, Borras E, et al. Association of fish consumption and mercury exposure during pregnancy with metabolic health and inflammatory biomarkers in children. *JAMA Netw Open*. 2020;3(3):e201007. doi:10.1001/jamanetworkopen.2020.1007

eMethods 1. Measurement of Mercury Levels During Pregnancy

eMethods 2. Measurement of Inflammatory Biomarkers and Adipokines in Children

eMethods 3. Covariates

eMethods 4. Structural Integrated Latent Variable Analysis

eTable 1. Description of Cohort-Specific Food Questionnaires Used to Assess Maternal Diet During Pregnancy

eTable 2. Comparison of Characteristics of the Study Population (N = 805) by Categories of Maternal Fish Consumption During Pregnancy

eTable 3. Associations of Fish Intake During Pregnancy and Maternal Mercury Levels With Metabolic Syndrome Score Among Children Aged 8 Years in HELIX after Stratifying for Child Sex, Maternal Education Level, and Gestational Diabetes Status

eTable 4. Associations of Fish Intake During Pregnancy and Maternal Mercury Levels With Metabolic Syndrome Score Among Children Aged 8 Years in HELIX (N = 805) After Further Adjusting for Gestational Weight Gain, Breastfeeding, Child Sedentary Behavior, Child Mercury Concentration, and a Number of Maternal and Child Food Indicators of Diet Quality

eTable 5. Associations of Fish Intake During Pregnancy and Maternal Mercury Levels With Metabolic Syndrome Score Among Children Aged 8 Years in HELIX (N = 805) After Further Adjusting for Maternal Plasma Levels of Polychlorinated Biphenyls, Dichlorodiphenyldichloroethylene, and Arsenic

eTable 6. Associations of Fish Intake During Pregnancy and Maternal Mercury Concentrations With Protein Levels Among Children Aged 8 Years in HELIX (N = 805)

eTable 7. Distribution of Exposure and Proteins in Subgroups From the Latent Integrated Analysis Defined With Individuals Assigned to the Most Likely Cluster

eFigure 1. Directed Acyclic Graph on the Association of Maternal Fish Intake During Pregnancy and Metabolic Health in Children

eFigure 2. Associations of Fish Intake During Pregnancy and Maternal Mercury Levels With Metabolic Syndrome Score Among Children Aged 8 Years in HELIX After Omitting 1 Cohort at a Time

eReferences.

This supplementary material has been provided by the authors to give readers additional information about their work.

© 2020 Stratakis N et al. *JAMA Network Open*.

eMethods 1. Measurement of Mercury Levels During Pregnancy

Maternal blood samples were collected in mid-pregnancy for MoBa (18.7 (0.9) weeks) and RHEA (14.1 (3.7) weeks), in late pregnancy for BiB (26.6 (1.4) weeks) and EDEN (26.1 (1.2) weeks), while, for INMA, we collected cord blood samples at delivery. The HELIX project measured whole blood mercury concentration in MoBa, RHEA, BiB, and EDEN cohorts in the same laboratory (ALS Scandinavia, Sweden) using biological samples stored in the cohort biobanks and utilized existing cord blood mercury measurements for INMA performed in the Public Health Laboratory in Alava (Spain). Mercury concentration in whole blood samples was determined using inductively coupled plasma mass spectrometry. Mercury in cord blood was measured using thermal decomposition, amalgamation and atomic absorption spectrometry. Details of the analytical procedures used for the HELIX project have been described previously.[1]

© 2020 Stratakis N et al. *JAMA Network Open*.

eMethods 2. Measurement of Inflammatory Biomarkers and Adipokines in Childhood

Blood samples were randomized and blocked by cohort prior to measurement to ensure a representation of each cohort in each plate (batch). For protein quantification, an 8-point calibration curve per plate was performed with protein standards provided in the Luminex kit and following the procedures described in the standard procedures described by the vendor. Commercial heat inactivated, sterile-filtered plasma from human male AB plasma (Sigma Cat #. H3667) was used as constant controls to control for intra- and inter-plate variability. Four control samples were added per plate. Raw intensities obtained with the xMAP and Luminex system for each sample were converted to pg/mL using the calculated standard curves of each plate and accounting for the dilutions that were made prior measurement. The proportion of coefficients of variation (% CV) for each protein, estimated by plate and then averaged, ranged from 3.42% to 36%. For each protein, the LOD was determined and the lower and upper quantification limits (LOQ1 and LOQ2, respectively) were obtained from the calibration curves. Proteins were not used if 30% of samples were outside of the linear range of quantification. Protein data were log2-transformed to achieve normal distribution. Plate batch effect was then corrected by subtracting for each individual and each protein the difference between the overall protein average minus the plate specific protein average. Finally, values below LOQ1 and above LOQ2 were imputed using a truncated normal distribution using the truncdist R package.

© 2020 Stratakis N et al. *JAMA Network Open*.

eMethods 3. Covariates

Potential confounding variables and effect modifiers were harmonized among the cohorts. Information on pre- pregnancy BMI (in kg/m2), gestational diabetes status (yes, no; diagnosis based on universal screening [BiB, EDEN, RHEA and MoBa] or selective screening among high-risk women [INMA]), maternal age at birth (in years), and parity (primiparous, multiparous) was obtained through interviews and medical records. Information on parental education (cohort-specific definition of low, middle, high), breastfeeding (yes, no), child ethnicity (Caucasian, Asian, Other; mother-reported), child sedentary behavior (in minutes/week), and child consumption of fast-food, sugar-sweetened beverages, sweets, and fish intake (in times/week) was collected with interviews or self-administered questionnaires. Moreover, we assessed levels of polychlorinated biphenyls (PCBs; the 118, 138, 153, 170, and 180 congeners), dichlorodiphenyldichloroethylene (DDE) and arsenic in maternal serum or plasma samples and mercury levels in child whole blood samples as described previously.[1]

© 2020 Stratakis N et al. *JAMA Network Open*.

eMethods 4. Structural Integrated Latent Variable Analysis

A model describing the MetS score (Y) as a function of cluster, C is $(\mu_Y) = \gamma_0 + \gamma_S C$, where γ_S represents the effect of each estimated cluster C, on the outcome Y. The clusters are defined as a multivariate normal model, $M \sim MVN(C\theta, \Sigma)$, where θ represents mean differences of the protein levels M by clusters, and Σ represents the correlation of protein profiles by C. This component of the model is similar to the iCluster approach. The estimation of the k clusters, C, follows a multinomial model with a linear predictor as a function of the exposures X, giving Pr(C=k | X), with corresponding effect estimates. In this model we include both fish consumption and mercury as main effects, and do not include an interaction term between these two factors. However, as both factors are in the linear predictor they both impact the probability of each estimated latent cluster. To estimate the overall integrated model, we treat cluster estimation as a missing data problem, where we use the Expectation-Maximization (E-M) algorithm for estimation with the E-step using the conditional probabilities, and M-step using the expected log-likelihood from the E-step maximized with respect to the parameters.[2] Although this can include the outcome in a supervised clustering approach, in this analysis, we define the clusters from the protein profiles and exposure variables only and then test the association of these clusters to MetS score. For the estimation of the number of latent clusters, we use Bayesian Information Criteria.

© 2020 Stratakis N et al. *JAMA Network Open*.

eTable 1. Description of Cohort-Specific Food Questionnaires Used to Assess Maternal Diet During Pregnancy

	Time of Administration	Number of Food Items	Period Covered	Type of Validation
BiB	26th–28th week	54	2nd trimester	-
EDEN	Few days after delivery	137	3rd trimester	4 x 24-h dietary recalls
MoBa	17th-18th week	250	1st half	1 x 4-day weighed food diary & urinary and blood biomarkers of nutrients and/or major food groups
Rhea	14th–18th week	250	1st half	3 x 24-h dietary recalls
INMA	10th–13th week	101	1st trimester	Blood biomarkers of nutrients & 4 x 1 week food records

Abbreviations: BiB, Born in Bradford cohort; EDEN, the Étude des Déterminants pré et postnatals du développement et de la santé de l'Enfant study; INMA, INfancia y Medio Ambiente cohort; MoBa, Norwegian Mother, Father and Child Cohort Study; RHEA, Rhea Mother Child Cohort study.

© 2020 Stratakis N et al. *JAMA Network Open*.

eTable 2. Comparison of Characteristics of the Study Population (N = 805) by Categories of Maternal Fish Consumption During Pregnancy

	Fish Consumption During Pregnancy		
	Low (<1 times/week) N= 117	Moderate (≥1 but ≤3 times/week) N= 317	High (>3 times/week) N= 371
**Parental characteristics**			
Maternal age, mean (SD), years	30.6 (4.8)[a]	30.9 (5)[a]	31.9 (4.2)[b]
Missing, n (%)	1 (<1)	1 (<1)	1 (<1)
Maternal pre-pregnancy BMI, mean (SD), kg/m^2	24.4 (4.7)[a]	24.2 (4.3)[a]	23.7 (4.3)[a]
Normal weight (<25kg/m^2), n (%)	78 (67)[a]	208 (66)[a]	266 (72)[a]
Overweight (≥25 kg/m^2), n (%)	36 (31)[a]	105 (33)[a]	102 (27)[a]
Missing, n (%)	3 (3)	4 (1)	3 (<1)
Maternal smoking in pregnancy, n (%)			
No	94 (80)[a]	259 (82)[a]	309 (83)[a]
Yes	21 (18)[a]	54 (17)[a]	56 (15)[a]
Missing	2 (2)	4 (1)	6 (2)
Gestational diabetes, n (%)			
No	98 (83)[a]	163 (51)[a]	123 (33)[a]
Yes	10 (9)[a]	22 (7)[a]	9 (3)[a]
Missing	9 (8)	132 (42)	239 (64)
Parity, n (%)			
Primiparous	53 (45) [a]	130 (41)[b]	185 (50)[b]
Multiparous	62 (53) [a]	185 (58)[b]	185 (50)[b]
Missing	2 (2)	2 (<1)	1 (<1)
Maternal educational level, n (%)			
Low	4 (3)[a]	45 (14)[b]	57 (15)[b]
Medium	60 (51)[a]	109 (34)[b]	114 (31)[b]
High	51 (44)[a]	155 (49)[b]	194 (52)[b]
Missing	2 (2)	8 (3)	6 (2)
Paternal educational level, n (%)			
Low	12 (10)[a]	47 (15)[b]	71 (19)[b]
Medium	61 (52)[a]	114 (36)[b]	119 (32)[b]
High	40 (34)[a]	123 (39)[b]	168 (45)[b]
Missing	4 (3)	33 (10)	13 (4)
Maternal mercury ≥3.5 µg/L, n (%)	36 (31)[a]	79 (25)[a]	155 (42)[b]
**Child characteristics**			
Age at assessment, mean (SD), years	7.7 (1.8)[a]	8.4 (1.8)[b]	8.6 (1.1)[b]
Sex, n (%)			
Male	70 (60)[a]	183 (58)[a]	200 (54)[a]
Female	47 (40)[a]	134 (42)[a]	171 (46)[a]
Birthweight, mean (SD), grams	3268 (472)[a]	3350 (479)[a]	3371 (498)[a]
Missing, n (%)	1 (<1)	-	-
Gestational age, mean (SD), weeks	38.7 (1.8)[a]	39.6 (1.7)[b]	40 (1.6)[c]
Missing, n (%)	1 (<1)	-	-
Ethnicity, n (%)			
Caucasian	117 (100)[a]	276 (87)[b]	341 (92)[c]
Asian	-	28 (9)[b]	27 (7)[c]
Other	-	13 (4)[b]	3 (1)[c]

[a-c]Values indicate significant differences between the categories of maternal fish intake at an alpha level of .05.

© 2020 Stratakis N et al. *JAMA Network Open*.

eTable 3. Associations of Fish Intake During Pregnancy and Maternal Mercury Levels With Metabolic Syndrome Score Among Children Aged 8 Years in HELIX After Stratifying for Child Sex, Maternal Education Level, and Gestational Diabetes Status

	**Metabolic syndrome score**		
	**Estimate (95% CI)**		**P-interaction**
Sex	Boys (n=453)	Girls (n=352)	
Fish intake, times/week			0.44
<1	Ref.	Ref.	
≥1 but ≤3	-0.85 (-1.54, -0.16)	-1.02 (-1.91, -0.14)	
>3	-0.70 (-1.50, 0.10)	-0.62 (-1.63, 0.38)	
Mercury, log2 μg/L	0.03 (-0.20, 0.25)	0.30 (0.06, 0.55)	0.12
Maternal education	Low/medium (n=389)	High (n=400)	
Fish intake, times/week			0.61
<1	Ref.	Ref.	
≥1 but ≤3	-1.06 (-1.87, -0.25)	-0.84 (-1.56, -0.11)	
>3	-0.82 (-1.80, 0.16)	-0.59 (-1.40, 0.22)	
Mercury, log2 μg/L	0.18 (-0.06, 0.43)	0.21 (-0.01, 0.44)	0.90
Gestational diabetes	No (n=389)	Yes (n=41)	
Fish intake, times/week			0.85
<1	Ref.	Ref.	
≥1 but ≤3	-1.03 (-1.66, -0.4)	-2.41 (-4.38, -0.43)	
>3	-0.12 (-0.96, 0.73)	-2.36 (-4.96, 0.23)	
Mercury, log2 μg/L	0.18 (-0.06, 0.41)	0.24 (-0.66, 1.14)	0.35

Abbreviations: MetS, metabolic syndrome.

[a]The MetS score is expressed in standard deviations and was derived using z-scores for waist circumference, systolic and diastolic blood pressure, high-density lipoprotein cholesterol, triglycerides, and insulin, as described in the Methods section. Estimates are beta coefficients (95% CI) calculated by a linear regression model mutually including maternal fish intake and mercury levels and further adjusted for cohort, maternal age, maternal pre-pregnancy BMI, maternal education (for models not stratified by this variable), paternal education, parity, and child ethnicity.

[b] P for interaction calculated by including a cross-product term between the potential effect modifier and maternal fish intake or mercury levels.

© 2020 Stratakis N et al. *JAMA Network Open*.

eTable 4. Associations of Fish Intake During Pregnancy and Maternal Mercury Levels With Metabolic Syndrome Score Among Children Aged 8 Years in HELIX (N = 805) After Further Adjusting for Gestational Weight Gain, Breastfeeding, Child Sedentary Behavior, Child Mercury Concentration, and a Number of Maternal and Child Food Indicators of Diet Quality

	**MetS Score[a]**	
	**Estimate (95% CI)**	***P* Value**
Fish intake, times/week		
<1	Ref.	
≥1 but ≤3	-0.97 (-1.51, -0.43)	<0.001
>3	-0.75 (-1.38, -0.11)	0.02
Mercury, log2 μg/L	0.19 (0.03, 0.36)	0.02

Abbreviations: MetS, metabolic syndrome.

[a]The MetS score is expressed in standard deviations and was derived using z-scores for waist circumference, systolic and diastolic blood pressure, high-density lipoprotein cholesterol, triglycerides, and insulin, as described in the Methods section. Estimates are beta coefficients (95% CI) calculated by a linear regression model mutually including maternal fish intake and mercury levels and further adjusted for cohort, maternal age, maternal pre-pregnancy BMI, gestational weight gain, maternal consumption of fruits and vegetables, cereals, and fast food during pregnancy, parental education, parity, child ethnicity, breastfeeding, child sedentary behavior, child mercury levels, and child consumption of total sweets, sugar-sweetened beverages, fast food, and fish. Mean variance inflation factor was 2.5, with all variance inflation factors for the predictors in the model being <10.

© 2020 Stratakis N et al. *JAMA Network Open*.

eTable 5. Associations of Fish Intake During Pregnancy and Maternal Mercury Levels With Metabolic Syndrome Score Among Children Aged 8 Years in HELIX (N = 805) After Further Adjusting for Maternal Plasma Levels of Polychlorinated Biphenyls, Dichlorodiphenyldichloroethylene, and Arsenic

	**MetS Score[a]**	
	**Estimate (95% CI)**	***P* Value**
Fish intake, times/week		
<1	Ref.	
≥1 but ≤3	-0.97 (-1.51, -0.43)	<0.001
>3	-0.71 (-1.34, -0.08)	0.03
Mercury, log2 µg/L	0.18 (0.01, 0.36)	0.04

Abbreviations: MetS, metabolic syndrome

[a]The MetS score is expressed in standard deviations and was derived using z-scores for waist circumference, systolic and diastolic blood pressure, high-density lipoprotein cholesterol, triglycerides, and insulin, as described in the Methods section. Estimates are beta coefficients (95% CI) calculated by a linear regression model mutually including maternal fish intake and mercury levels and further adjusted for maternal plasma levels of polychlorinated biphenyls (the sum of 118, 138, 153, 170, and 180 congeners), dichlorodiphenyldichloroethylene and arsenic, maternal age, maternal pre-pregnancy BMI, parental education, parity, child ethnicity, and cohort. Mean variance inflation factor was 2.9, with all variance inflation factors for the predictors in the model being <10.

© 2020 Stratakis N et al. *JAMA Network Open*.

eTable 6. Associations of Fish Intake During Pregnancy and Maternal Mercury Concentrations With Protein Levels Among Children Aged 8 Years in HELIX (N = 805)

	Fish Intake (≥1 time/week)			Mercury (log2 mg/L)	
	**Percent hange (95% CI)**	***P***		**Percent change (95% CI)**	***P***
**JAK-STAT pathway**					
EGF	10.97 (-20.19, 54.28)	0.54		5.06 (-4.91, 16.07)	0.33
IFN-alpha	-0.91 (-15.01, 15.53)	0.91		1.79 (-2.83, 6.63)	0.45
IFN-gamma	-3.53 (-22.82, 20.59)	0.75		3.71 (-3.06, 10.95)	0.29
IL-2	-2.69 (-22.01, 21.43)	0.81		-2.39 (-8.71, 4.37)	0.48
IL-2R	6.15 (-6.69, 20.76)	0.36		-2.88 (-6.59, 0.98)	0.14
IL-4	5.49 (-9.76, 23.32)	0.5		1.56 (-3.13, 6.47)	0.52
IL-5	-1.40 (-19.16, 20.26)	0.89		-0.3 (-6.11, 5.88)	0.92
IL-6	-16.24 (-24.89, -6.6)	0.001		2.5 (-0.82, 5.94)	0.14
IL-10	3.13 (-19.39, 31.94)	0.81		0.69 (-6.54, 8.48)	0.86
IL-12	1.21 (-8.59, 12.08)	0.82		1.63 (-1.45, 4.82)	0.3
IL-13	4.26 (-11.52, 22.87)	0.62		-2.31 (-7.04, 2.67)	0.36
IL-15	-12.78 (-36.24, 19.31)	0.39		6.98 (-2.69, 17.62)	0.16
Leptin	-7.10 (-23.26, 12.46)	0.45		2.66 (-3.11, 8.77)	0.37
Pathway effect	0.39 (-5.18, 6.29)	0.89		1.17 (-0.69, 3.05)	0.21
**Adipocytokine pathway**					
Adiponectin	-11.37 (-18.69, -3.4)	0.01		0.16 (-2.42, 2.8)	0.90
Leptin	-7.10 (-23.26, 12.46)	0.45		2.66 (-3.11, 8.77)	0.37
TNF-alpha	-5.71 (-10.55, -0.60)	0.03		0.17 (-1.41, 1.78)	0.83
Pathway effect	-5.76 (-10.88, -0.35)	0.04		0.39 (-1.41, 2.22)	0.66
**NF-kappa B pathway**					
BAFF	-1.61 (-6.3, 3.33)	0.52		-0.35 (-1.81, 1.14)	0.64
IL-1beta	-16.73 (-27.87, -3.86)	0.01		4.29 (-0.15, 8.92)	0.06
IL-8	-1.11 (-6.46, 4.56)	0.7		-0.22 (-1.88, 1.48)	0.80
MIP-1beta	3.39 (-7.96, 16.14)	0.57		-0.57 (-4, 2.99)	0.75
TNF-alpha	-5.71 (-10.55, -0.60)	0.03		0.17 (-1.41, 1.78)	0.83
Pathway effect	0.79 (-3.47, 5.24)	0.71		0.14 (-1.25, 1.55)	0.84
**Chemokine pathway**					
IL-8	-1.11 (-6.46, 4.56)	0.7		-0.22 (-1.88, 1.48)	0.8
MCP-1	-6.69 (-13.07, 0.15)	0.06		-1.14 (-3.24, 1)	0.29
MIG	7.17 (-5.41, 21.42)	0.28		0.72 (-3.01, 4.59)	0.71
MIP-1alpha	-1.96 (-11.24, 8.29)	0.7		1.65 (-1.36, 4.76)	0.29
MIP-1beta	3.39 (-7.96, 16.14)	0.57		-0.57 (-4, 2.99)	0.75
Pathway effect	0.13 (-4.59, 5.08)	0.96		0.01 (-1.54, 1.59)	0.99
**Cholesterol metabolism**					
apoA1	-5.3 (-12.41, 2.38)	0.17		0.36 (-1.98, 2.75)	0.77
apoB	-1.55 (-7.41, 4.67)	0.62		-0.87 (-2.7, 0.98)	0.35
apoE	-9.68 (-18.66, 0.3)	0.06		-1.19 (-4.27, 1.99)	0.46
Pathway effect	-2.09 (-7.45, 3.58)	0.45		-0.29 (-2.09, 1.55)	0.75

Abbreviations: apo, apolipoprotein; BAFF, B cell activating factor of the TNF family; EGF, epidermal growth factor; IFN, interferon; IL, interleukin; MCP, methyl-accepting chemotaxis protein; MIG, C-X-C motif chemokine 9; MIP, Macrophage inflammatory protein; TNF-alpha, tumor necrosis factor alpha.

Effect estimates are percent changes (95% CI) in protein levels (in log2 pg/ml) estimated after using linear regression models mutually including maternal fish intake and mercury concentrations and being further adjusted for maternal age, maternal pre-pregnancy BMI, parental education, parity, child ethnicity, child sex, child age and cohort. The reference category for fish intake is less than 1 time/week. A Bonferroni correction for multiple testing would result in a significance threshold of a=0.001.

© 2020 Stratakis N et al. *JAMA Network Open.*

eTable 7. Distribution of Exposure and Proteins in Subgroups From the Latent Integrated Analysis Defined With Individuals Assigned to the Most Likely Cluster[a]

	**Cluster 1 Low MetS Score (N=635)**	**Cluster 2 High MetS Score (N=170)**
**Distribution by cohort, n (%)**		
BIB, UK	96 (15.1)	8 (4.7)
EDEN, France	96 (15.1)	47 (27.6)
INMA, Spain	140 (22.0)	64 (37.6)
MoBa, Norway	191 (30.1)	20 (11.8)
RHEA, Greece	112 (17.7)	31 (18.2)
**Exposures**		
Fish intake during pregnancy, n (%)		
<1 times/week	81 (12.8)	36 (21.2)
≥1 but ≤3 times/week	261 (41.1)	56 (32.9)
>3 times/week	293 (46.1)	78 (45.9)
Mercury, median (25th,75th), µg/L	2.3 (1.4, 3.9)	3.4 (2.0, 5.5)
**Metabolic risk factors**		
WC, mean (SD), cm	56.5 (4.7)	69.7 (7.6)
HDL, mean (SD), mg/dL	61.4 (12.6)	55.6 (10.9)
TG, median (25th,75th), mg/dL	70.9 (55.8, 93.0)	93.0 (70.2, 125.8)
Insulin, median (25th,75th), µU/mL	5.0 (4.0, 7.1)	8.7 (6.0, 12.5)
BP, mean (SD), mm Hg		
Systolic	98.9 (10.2)	106.1 (10.1)
Diastolic	57.4 (9.5)	60.9 (8.5)
**Proteins**		
Adiponectin, median (25th,75th), µg/mL	25.1 (19.6, 29.7)	23.5 (16.9, 28.9)
Leptin, median (25th,75th), ng/mL	1.0 (0.5, 1.7)	1.9 (0.8, 3.5)
TNF-alpha, median (25th,75th), pg/mL	30.2 (26.3, 32.3)	32.1 (30.2, 36.5)
IL-1beta, median (25th,75th), pg/mL	9.5 (7.3, 12.7)	34.8 (25.8, 51.7)
IL-6, median (25th,75th), pg/mL	9.6 (7.8, 11.7)	23.2 (17.9, 30.9)

Abbreviations: BP, blood pressure, HDL, high-density lipoprotein cholesterol; IL, interleukin; MetS, metabolic syndrome; TG, triglycerides; WC, waist circumference.
[a]Posterior probability cutoff: 0.5.

© 2020 Stratakis N et al. *JAMA Network Open*.

eFigure 1. Directed Acyclic Graph on the Association of Maternal Fish Intake During Pregnancy and Metabolic Health in Children

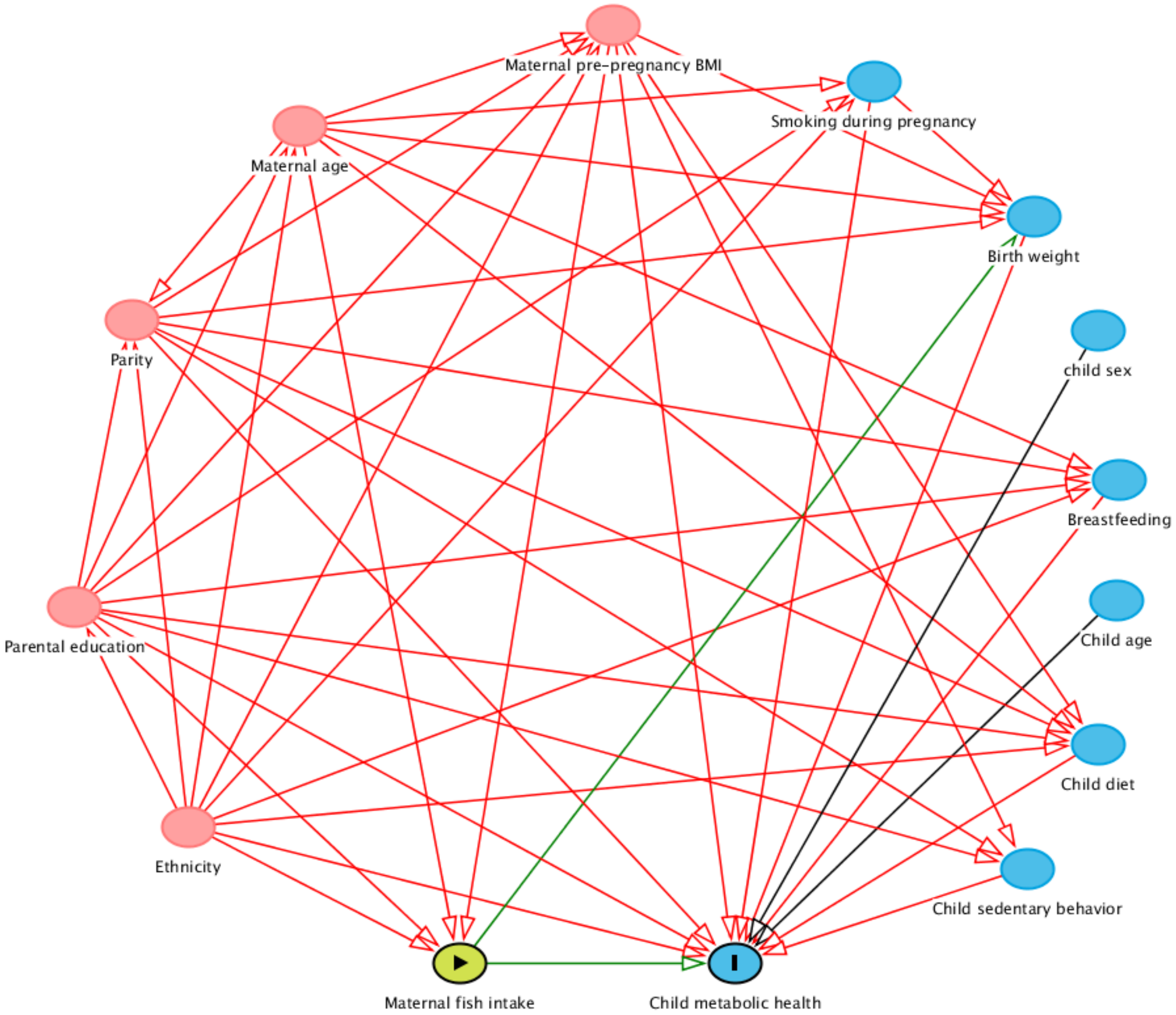

Red nodes represent ancestors of the exposure and outcome; blue nodes represent ancestors of the outcome. Graph was constructed using DAGitty version 2.2.[3]

© 2020 Stratakis N et al. *JAMA Network Open*.

eFigure 2. Associations of Fish Intake During Pregnancy and Maternal Mercury Levels With Metabolic Syndrome Score Among Children Aged 8 Years in HELIX After Omitting 1 Cohort at a Time

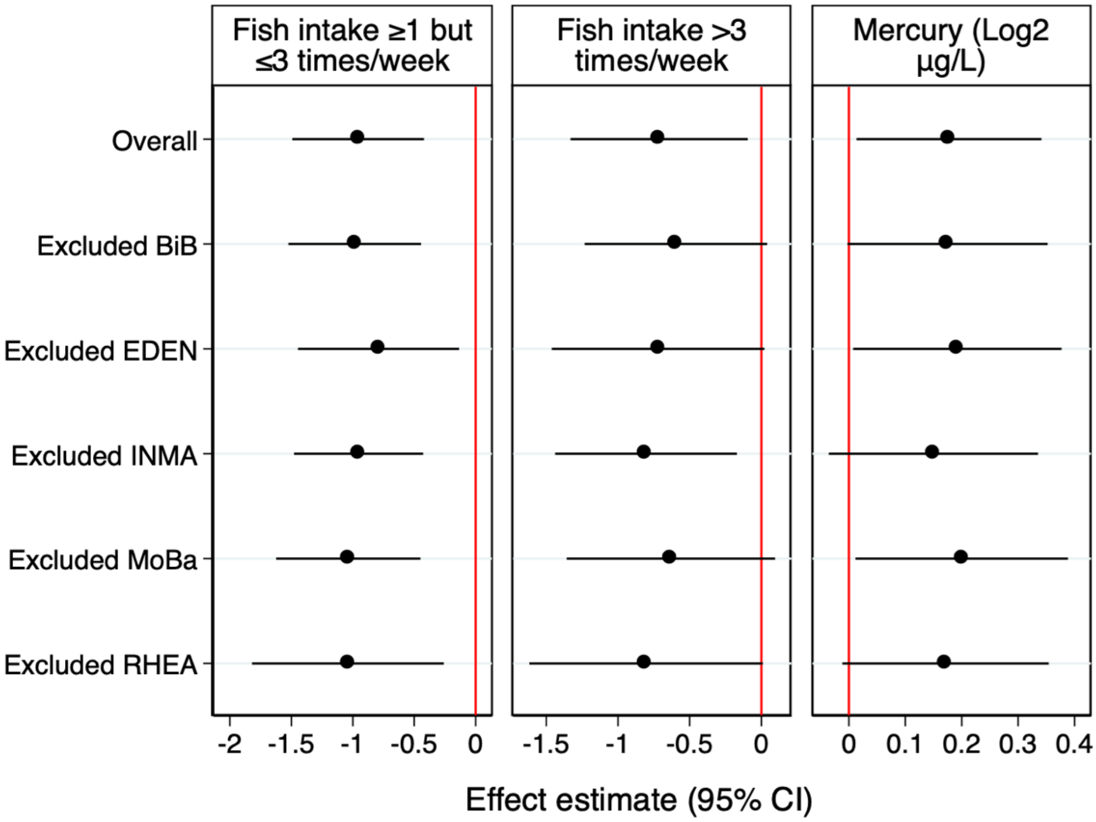

The metabolic syndrome score is expressed in standard deviations and was derived using z-scores for waist circumference, systolic and diastolic blood pressure, high-density lipoprotein cholesterol, triglycerides, and insulin, as described in the Methods section. Effect estimates represent beta coefficients (circles) and 95% CIs (error bars) calculated by linear regression models mutually including maternal fish intake and mercury concentration and further adjusted for maternal age, maternal pre-pregnancy BMI, parental education, parity, child ethnicity, and cohort. For fish intake, the reference category is less than 1 time/week. BiB, Born in Bradford cohort; EDEN, the Étude des Déterminants pré et postnatals du développement et de la santé de l'Enfant study; INMA, INfancia y Medio Ambiente cohort; MoBa, Norwegian Mother, Father and Child Cohort Study; RHEA, Rhea Mother Child Cohort study.

© 2020 Stratakis N et al. *JAMA Network Open*.

eReferences

1. Haug LS, Sakhi AK, Cequier E, et al. In-utero and childhood chemical exposome in six European mother-child cohorts. *Environ Int.* 2018;121(pt 1):751-763.
2. Peng C, Wang J, Asante I, et al. A latent unknown clustering integrating multi-omics data (LUCID) with phenotypic traits. Bioinformatics [published online August 29, 2019].
3. Textor J, Hardt J, Knuppel S. DAGitty: a graphical tool for analyzing causal diagrams. *Epidemiology.* 2011;22(5):745.

© 2020 Stratakis N et al. *JAMA Network Open*.
